# A Hot Topic: A Climate-Focused Track for Infectious Diseases Fellowship

**DOI:** 10.1093/ofid/ofaf080

**Published:** 2024-11-14

**Authors:** Noah Rosenberg, Satchit Balsari, Caleb Dresser, Douglas Krakower, Wendy Stead

**Affiliations:** Department of Infectious Diseases, Beth Israel Deaconess Medical Center, Boston, Massachusetts, USA; Department of Emergency Medicine, Beth Israel Deaconess Medical Center, Boston, Massachusetts, USA; Department of Global Health, Harvard T. H. Chan School of Public Health, Boston, Massachusetts, USA; Department of Emergency Medicine, Beth Israel Deaconess Medical Center, Boston, Massachusetts, USA; Department of Environmental Health, Harvard T. H. Chan School of Public Health, Boston, Massachusetts, USA; Department of Infectious Diseases, Beth Israel Deaconess Medical Center, Boston, Massachusetts, USA; Department of Infectious Diseases, Beth Israel Deaconess Medical Center, Boston, Massachusetts, USA

**Keywords:** climate change, infectious diseases, medical education

## Abstract

Climate change has substantial implications for infectious diseases. The infectious diseases community must rapidly expand its understanding of these dynamics; formal training for physicians is urgently needed. Here, we describe the learning objectives, educational strategy, and implementation of a pilot Climate Change training pathway within an infectious diseases fellowship.

## Teaser

With increasing impacts of climate change on infectious diseases, there is now a need for formalized education for clinicians in these intersecting fields. We describe the learning objectives and planned implementation of a climate-focused track within an infectious diseases fellowship.

Climate change is a threat with adverse health effects that have been increasing in severity and expanding in scope over the recent decades [1]. These effects span many different aspects of disease burden and severity, ranging from heat-related illness to food insecurity [2]. Populations at risk are seeing accelerating and disproportionate effects of climate change, while geographic regions previously thought to be unaffected now feel direct and indirect effects, leaving politicians, city planners, environmental agencies, and healthcare professionals underprepared to address these impacts [3].

There is an increasing concern regarding the effects of climate change on infectious diseases [4, 5]. These effects can be categorized into diseases and pathogens directly affected by climate change, epidemiologic shifts where pathogens and people are brought into closer proximity, and effects on the susceptibility of hosts to these infectious diseases [6].

Given these effects, there is an increasing need to better prepare physicians to recognize the consequences of climate change effects on their clinical practice. However, previous education on this topic for infectious diseases trainees has been predominantly informal, through conference sessions, lectures, short courses, and published reviews [7, 8].

Formalized educational pathways to educate physicians on the health impacts of climate change may be more effective than ad hoc approaches, as many health professionals state they feel a responsibility to act on climate change but lack specific training to do so. Several Graduate Medical Education (GME) and non-GME fellowships in climate change and health have been established, improving participating physicians’ understanding of the impacts of climate on health and strategies to address climate effects, helping them to become leaders in this field [9–16].

In 2019, the Department of Emergency Medicine at Beth Israel Deaconess Medical Center (BIDMC) established a formal climate-focused training pathway for emergency medicine physicians that integrates clinical practice, scholarship, and education through climate-oriented curricular content and research training.

Given the early successes of this unique fellowship training program, in order to address the escalating need for infectious diseases physician leaders in research, policy, education, and clinical care at the intersection of climate change and infectious diseases, the Division of Infectious Diseases at BIDMC is partnering with the Climate and Human Health Fellowship in the Department of Emergency Medicine to establish a novel Climate Change and Infectious Diseases training pathway.

## INFECTIOUS DISEASES CLINICAL FELLOWSHIP: CLIMATE HEALTH PATHWAY

### Overview

The Climate Health and Infectious Diseases pathway is being piloted in 2024 as a unique scholarly track within the Infectious Diseases Fellowship Training Program at BIDMC.

### Goals and Objectives

The goal of this pathway is to train infectious diseases fellows to become well-informed clinician-scholars in the intersection of infectious diseases and climate health, by providing a comprehensive educational experience that includes climate-related infectious diseases curricular content, clinical experience, policy, and research (Table 1) [17].

**Table 1.** Content Knowledge Areas

Content Area	Learning Objectives
Climate change mechanisms	Describe the concept of anthropogenic climate change Explain the role of greenhouse gases and the effect of human activity on these emissions
Climate-responsive hazards	Relate climate change to extreme weather events including heatwaves, hurricanes, heavy rainfall, and floods, on shorter timescales; to longer-term impacts on land use, sea level rise, and drought; and to biome shifts, marine impacts, and other aspects of global change Identify individual- and systems-level responses to these hazards
Mechanisms linking climate change and infectious diseases	Distinguish the various mechanisms that connect climate change with exacerbation, change, and further impacts on the pathophysiology of infectious diseases and their spread Assess the level of evidence supporting each of these connections
Infectious diseases in the context of climate change	Examining pathways (and evidence thereof) for long- and short-term climate change impact on vectors, animal reservoirs, and other sources of pathogens, and their possible implications for population health Describe the impacts of climate change on the epidemiology of vector-borne diseases, fungal diseases, water- and food-borne diseases, and zoonotic diseases and the possible implications for training of health systems, preparedness, and resource utilization Develop a framework for incorporating understanding and a clinically focused approach of the above pathways and impacts of climate change to address patient care in a changing environment Understand the impacts of climate change on infectious diseases in vulnerable populations through direct mechanisms as well as through secondary impacts such as food insecurity and climate migration
Infectious diseases control and surveillance in a changing climate	Assess current surveillance methods for infectious diseases and how they may predict the effects of climate change on a disease Learn how climate change affects current surveillance methods and develop strategies to adapt surveillance methods to assess the impact of climate change Gain an understanding of the role an individual clinician plays in contributing to surveillance of outbreaks
Antimicrobial resistance implications of climate change	Identify mechanisms by which climate change may affect antimicrobial resistance Assess the level of evidence connecting climate change with antimicrobial resistance patterns
Sustainable practice of clinical infectious diseases and infection prevention	Understand current efforts in sustainability within infectious diseases Identify and evaluate sources of waste in infectious diseases and how to improve sustainability of the methods
Climate, health policy, and governance	Assess threats and effects of climate change on healthcare systems and understand how policy may address this Develop skills in communicating climate-focused concerns with policy-makers and stakeholders
Engagement with public media	Identify key points to convey during an interview Compose responses to questions about climate change and its health impacts during mock interviews as well as with journalists, focusing on conveying scientific validity and urgency.
Science communication	Employ verbal and visual approaches to communicate key points during public speaking with academic or medical audiences as well as public audiences Evaluate the response of audience members, and revise delivery style in response to audience cues

#### Clinical Education in Infectious Diseases and Climate Change

This track aims to ensure fellows learn how clinical infectious diseases intersect with climate change. Fellows will learn the impacts of climate change and environmental hazards on health and healthcare delivery. There will be a focus on the mechanisms by which climate change affects vector-borne, zoonotic, water-borne, food-borne, and respiratory diseases; impacts of extreme weather events on pathogen behavior; and the repercussions of rising temperatures on antimicrobial resistance patterns. Additionally, there will be opportunities to explore how infectious diseases practices and healthcare-related waste exacerbate climate change and the role of sustainability measures within infectious diseases. Fellows will be equipped with the tools to become diagnosticians with a climate-focused mindset, understanding how to incorporate their knowledge of climate hazards into their clinical decision-making, and provide support as clinicians to further the understanding of climate impacts on infectious diseases.

Learning will occur through complementary modalities. Foundational learning regarding climate change and its impacts on human health and disease will occur through participation in interactive didactics, a guided reading list, and completion of online content. In addition, infectious diseases–focused sessions will provide opportunities to further solidify this knowledge [18]. In order to provide a variety of perspectives on these issues, sessions will be led by climate scientists, clinical faculty from the Climate and Human Health Fellowship, and infectious diseases faculty.

Fellows will apply their new knowledge in clinical practice as appropriate and will cultivate a climate-focused approach to their care of the patient throughout their training. Fellows will present clinical cases related to climate change at regularly scheduled infectious diseases clinical case conferences. In addition, rotations through infection prevention services will give fellows opportunities to learn about sustainable practices in infection control and further understand the role of the clinician in the surveillance and identification of climate-related changes such as outbreaks and travel-associated infections. A rotation with the antimicrobial stewardship service will provide opportunities to learn about efforts at sustainability within stewardship and the impacts of climate change on antimicrobial resistance.

#### Public Health and Policy

Physicians graduating from this pathway will gain exposure to the history and status of policies related to climate change and their effects on healthcare delivery in infectious diseases, as well as how policies have the power to contribute to and address disparities in health-related outcomes due to climate change. Interactions with healthcare leaders as well as community and public health stakeholders will teach fellows to implement climate-oriented policies in routine care and about climate-related threats that healthcare systems may face. Through this, fellows will develop frameworks for organizational and community resilience and preparedness to address these threats [8, 19].

#### Scholarly Work and Research

Through guidance from external experts as well as affiliated faculty, fellows will participate in original research on a topic of their choosing. In addition, fellows will have opportunities to attend national and international conferences and policy forums on climate change and health to learn about, present on, and discuss developments in infectious diseases and climate change. There will also be opportunities to obtain education and experience in how to effectively communicate their scholarly work and disseminate information on health-related impacts of climate change to policy-makers, fellow scientists, and the general public, furthering general awareness and understanding, and inspiring action to address the problems at hand.

### Implementation

This training pathway is being implemented as part of the 2-year Clinical Infectious Diseases Fellowship at BIDMC. The fellowship's first year focuses on standard clinical training in inpatient and outpatient infectious diseases diagnosis and management; acquisition of core climate content knowledge will be achieved through participation in Climate and Human Health Fellowship didactics, journal clubs, and a guided reading list [20]. During the fellowship's second year, there is dedicated time for acquisition of additional content knowledge, relevant skills, and conduct of mentored scholarly research.

In addition to the knowledge and experience typically acquired during an infectious diseases fellowship, infectious diseases fellows in the Climate track will receive additional training in climate and health education, communication, and policy, following the Climate and Human Health Fellowship's model. Media engagement skills will be obtained through mock interviews and ongoing feedback from real-world interviews. Fellows will attend academic conferences and policy convenings, where they will be expected to engage with or observe the policymaking process when feasible and to educate others on their specific area of study. Public speaking for both academic and nonacademic audiences will be encouraged and constructively critiqued. Fellows will have opportunities to pursue part-time external experiential learning to gain firsthand knowledge of key organizations involved in addressing the health impacts of climate change; past Climate and Human Health fellows have participated in externships with nonprofit organizations, academic journals, and federal agencies.

Fellows will be expected to develop and implement original scholarly work that prepares them to serve as academic faculty or in a leadership position in a nonacademic scientific organization. Clinical research, epidemiology, and data analytic skills will be learned through available coursework and further supported via mentored research involving collaboration with data scientists and statisticians. Regular meetings with faculty leaders will provide individualized mentorship.

### Expectations, Evaluation, and Feedback

Trainees will be expected to demonstrate mastery of this content area by displaying competence in identifying, managing, and educating other trainees about infectious diseases impacted by climate change, successfully publishing scholarly works, and attending and participating at national and international meetings related to climate change and health. Fellows will be mentored by a multidisciplinary group of infectious diseases and emergency medicine faculty with climate expertise, with routine meetings to facilitate bidirectional feedback on didactics, self-guided learning, and clinical and scholarly experiences. This framework will ensure successful clinical training and scholarly productivity at the intersection of climate and infectious diseases by the completion of training.

## CONCLUSIONS

Few formal training opportunities exist to help infectious diseases physicians develop impactful careers at the nexus of these fields. To address this gap, we are piloting a climate change pathway within a clinical infectious diseases fellowship program at a major academic medical center. We hope this novel pathway will serve as a model for other climate change–focused pathways within infectious diseases and, perhaps, other subspecialty fellowships within internal medicine, addressing a need for a physician workforce prepared and passionate about addressing this urgent threat to our patients and communities.

## Notes

***Financial support.*** There was no direct funding for this study; however, funding of this climate track will occur through the Infectious Diseases Fellowship and Climate and Human Health Fellowship at Beth Israel Deaconess Medical Center.
